# The role of the adaptor molecule STING during *Schistosoma mansoni* infection

**DOI:** 10.1038/s41598-020-64788-6

**Published:** 2020-05-13

**Authors:** Cláudia Souza, Rodrigo C. O. Sanches, Natan R. G. Assis, Fábio V. Marinho, Fábio S. Mambelli, Suellen B. Morais, Enrico G. T. Gimenez, Erika S. Guimarães, Tiago B. R. Castro, Sergio C. Oliveira

**Affiliations:** 1 Departamento de Bioquímica e Imunologia, Instituto de Ciências Biológicas, Universidade Federal de Minas Gerais, Belo Horizonte, MG Brazil; 2 Departamento de Genética, Ecologia e Evolução, Instituto de Ciências Biológicas, Universidade Federal de Minas Gerais, Belo Horizonte, MG Brazil; 3 Instituto Nacional de Ciência e Tecnologia em Doenças Tropicais (INCT-DT), Conselho Nacional de Desenvolvimento Científico e Tecnológico, Ministério de Ciência Tecnologia e Inovação Salvador, Bahia, Brazil

**Keywords:** Parasitic infection, Immunology, Microbiology, Parasite host response

## Abstract

Schistosomiasis is a human parasitic disease responsible for serious consequences for public health, as well as severe socioeconomic impacts in developing countries. Here, we provide evidence that the adaptor molecule STING plays an important role in *Schistosoma mansoni* infection. *S. mansoni* DNA is sensed by cGAS leading to STING activation in murine embryonic fibroblasts (MEFs). Sting^−/−^ and C57BL/6 (WT) mice were infected with schistosome cercariae in order to assess parasite burden and liver pathology. Sting^−/−^ mice **showed** worm burden reduction but no change in the number of eggs or granuloma numbers and area when compared to WT animals. Immunologically, a significant increase in IFN-γ production by the spleen cells was observed in Sting^−/−^ animals. Surprisingly, Sting^−/−^ mice presented an elevated percentage of neutrophils in lungs, bronchoalveolar lavage, and spleens. Moreover, Sting^−/−^ neutrophils exhibited increased survival rate, but similar ability to kill schistosomula *in vitro* when stimulated with IFN-γ when compared to WT cells. Finally, microbiota composition was altered in Sting^−/−^ mice, revealing a more inflammatory profile when compared to WT animals. In conclusion, this study demonstrates that STING signaling pathway is important for *S. mansoni* DNA sensing and the lack of this adaptor molecule leads to enhanced resistance to infection.

## Introduction

Schistosomiasis is a neglected tropical disease caused by parasites of the genus *Schistosoma*^1^. The three major species infective to humans are *Schistosoma mansoni*, *S. japonicum*, and *S. haematobium*. It is considered the most prevalent neglected disease worldwide, after malaria^2^. There are approximately 779 million people living in areas with risk of infection and more than 250 million people infected with *Schistosoma* spp. worldwide^1^. The parasite *S. mansoni* is commonly found in Africa, in the Middle East, in South America, and in some of the Caribbean islands^3,4^. Infection occurs by the contact of human skin with freshwater contaminated by cercariae, previously released by the intermediate host snail^1^. After schistosome cercariae infect humans, they develop into adult worms in the host portal-vein mesenteric venous system. Eggs produced by female worms are mostly deposited in liver and intestine tissues. The characteristics of liver injury associated with *S. mansoni* infection are pronounced immunological and inflammatory responses caused by the soluble egg antigen released within eggs, leading to granuloma and subsequent fibrosis^4^. Hepatic fibrosis is the main cause of morbidity and mortality in humans with schistosome infection.

Inflammation is a crucial component in the development of liver fibrosis induced by schistosomes. Recently, several studies have suggested the importance of inflammasome receptors such as NLRP3 in schistosomes-mediated liver inflammation and fibrosis^5,6^. The innate immune system is known to be the first line of defense against invading pathogens, promoting and mediating recruitment of the adaptive immune response^7,8^. During infection, the host detects pathogen-associated molecular patterns (PAMP) through pattern-recognition receptors (PRR)^9^. Among them, DNA recognition is an evolutionarily conserved defense mechanism of imperative importance^10^. Several receptors are described as DNA sensors. The most studied of them are Toll-like receptor 9 (TLR9), Absent in Melanoma 2 (AIM2) and cyclic GMP-AMP Synthase (cGAS)^11^. cGAS detects cytosolic dsDNA through its binding to the sugar-phosphate backbone, regardless of the sequence^9^. This recognition promotes dimerization and the activation of cGAS, allowing ATP and GTP to access its catalytic cavity leading the synthesis of the second messenger cyclic GMP-AMP (2’3′-cGAMP)^12,13^. Then, 2’3′-cGAMP binds to the Stimulator of Interferon Genes (STING), leading to activation and nuclear translocation of transcription factors Interferon-Regulatory Factor 3 (IRF3) and Nuclear Factor κB (NF-κB). IRF3 and NF-κB promote subsequent expression of type I interferons (IFN), IFN-stimulated genes and cytokines/chemokines^11^. STING, also known as TMEM173, MITA, MPYS or ERIS, is an endoplasmic reticulum-located transmembrane protein that participates in several intracellular signaling pathways, such as DNA-dependent activator of IFN-Regulatory Factors (DAI), IFN-γ-Inducible Protein 16 (IFI16) and DEAD (Asp-Glu-Ala-Asp) Box Polypeptide 41 (DDX41)^8,14^. It has been widely described that STING plays an important role in cancer, autoimmune diseases, viral and bacterial infections. However, little is known about the implication of this pathway in the immune response against helminths^15^. *S. mansoni* larvae and adult worms migration in host tissues might induce cellular damage, leading to release of both endogenous and parasite DNA. There is some evidence that cargo molecules can assist extracellular DNA to get access to the intracellular space and trigger STING pathway^16^.

Here, we investigated the role of STING in the control of schistosomiasis infection and pathology induced by this disease. This study demonstrated for the first time that *S. mansoni* DNA is sensed by the cGAS/STING axis and lack of this signaling pathway renders mice more resistant to infection. Understanding the mechanisms involved in establishing schistosome infection may provide new approaches for therapeutic and prophylactic interventions.

## Results

### *Schistosoma mansoni* DNA activates the cGAS/STING pathway

**Figure 1:**
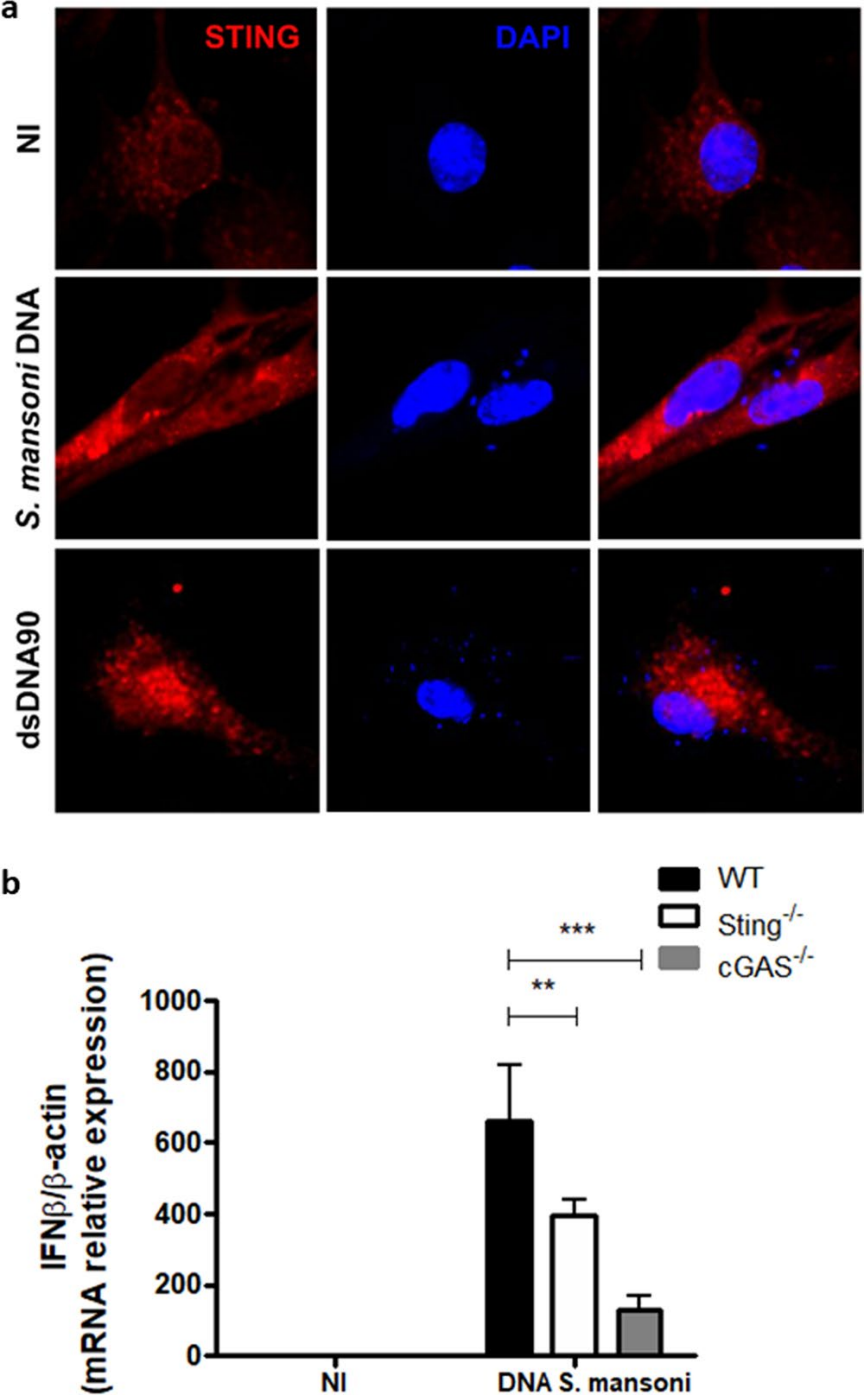
*Schistosoma mansoni* DNA recognition and STING activation in murine embryonic fibroblasts (MEFs). (**a**) Confocal microscopy of C57BL/6 (WT) MEFs stained with anti-STING (red) and DAPI (4,6-diamidino-2-phenylindole) (blue), transfected with Fugene only (NI) or transfected with 3 μg/mL of *S. mansoni* DNA or 3 μg/mL of STING-activating dsDNA (dsDNA90 base pairs) for 6 hours. (**b**) Quantitative reverse transcriptase–PCR (qRT–PCR) analysis of interferon-β (IFN-β) mRNA in WT and cGAS-/- and Sting^−/−^ MEFs transfected with Fugene only (NI, n = 3) or transfected with 3 μg/mL of *S. mansoni* DNA (n = 3) for 6 hours. NI represents transfected MEFs with Fugene only. (***) and (**) are used to demonstrate statistical differences with p < 0.001 and p < 0.01 compared to the WT MEFs, respectively. Two-Way ANOVA with Bonferroni adjustments were included for multiple comparisons.

DNA recognition by the immune system is a major strategy by which the host senses infection and initiates protective responses against pathogens^17,18^. In order to evaluate whether the STING signaling pathway is able to recognize *S. mansoni* DNA, C57BL/6 (WT) murine embryonic fibroblasts (MEFs) were transfected with the parasite DNA or dsDNA90 (positive control) for 6 hours. STING and DNA were then stained for confocal microscopy analysis. Figure 1a shows that STING was dispersed in the cytoplasm of the cells transfected with Fugene alone. However, when MEFs were transfected with *S. mansoni* DNA or dsDNA90, STING migrated from the cytoplasm to the perinuclear region of these cells, forming punctual aggregates (Fig. 1a). This is evidence that the parasite DNA was recognized, leading to STING activation. To further confirm this activation, *IFN-β* expression was measured in WT, and Sting^−/−^ and cGAS^−/−^ MEFs stimulated with *S. mansoni* DNA. Not detectable *IFN-β* expression was observed in cells transfected with Fugene alone. Furthermore, WT MEFs transfected with *S. mansoni* DNA produced high levels of *IFN-β* mRNA, indicating the parasite DNA was able to activate STING. In contrast, *IFN-β* expression was dramatically reduced in Sting^−/−^ and cGAS^−/−^ MEFs (Fig. 1b). These findings demonstrate that *S. mansoni* DNA sensing requires the cGAS/STING pathway.

### Lack of STING signaling renders mice more resistant to *S. mansoni* infection

**Figure 2:**
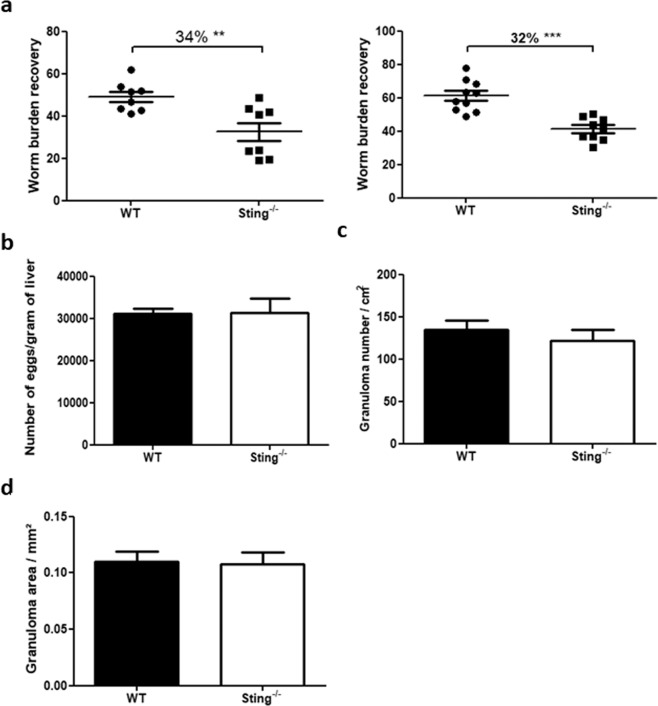
*In vivo* evaluation of worm burden and pathology in Sting^−/−^ mice. C57BL/6 (WT) and Sting^−/−^ mice (n = 10) were infected with 100 *S. mansoni* cercariae and, after 40 days of infection, the total number of worms recovered (**a**), number of eggs per gram of liver (**b**), number of granulomas per cm^2^ (**c**) and area of granulomas in mm^2^ (n = 10) (**d**) were evaluated. (**) and (***) are used to demonstrate statistical differences at p < 0.01 or p < 0.001 compared to the WT mice, respectively. For single comparisons, unpaired Student’s t-test was used.

Since *S. mansoni* DNA activates STING, we decided to evaluate the role of this adaptor molecule during *S. mansoni* infection. Sting^−/−^ and C57BL/6 (WT) mice were infected with 100 *S. mansoni* cercariae and worm burden evaluated. After 40 days of infection, Sting^−/−^ mice showed reduction of 34% and 32% in the number of recovered adult worms when compared to the control group (WT) (Fig. 2a). A similar resistant profile was also observed in cGAS^−/−^ animals (Supplementary Fig. S1). After perfusion, livers of Sting^−/−^ and WT mice were collected for egg counting and hepatic granuloma number and area assessment. Despite the reduced number of worms recovered in Sting^−/−^ mice, no significant differences were observed in both egg and granuloma counts (Fig. 2b,c). Moreover, STING deficiency does not affect hepatic granuloma maturation, represented by area measurement (Fig. 2d). It is possible that schistosome eggs can get trapped in other host tissues such as lung and intestinal wall, not investigated in this study. This may explain why we did not observe reduced number of eggs and granulomas in mice lacking STING.

### Humoral and cellular immune responses to *S. mansoni* in STING deficient mice

**Figure 3:**
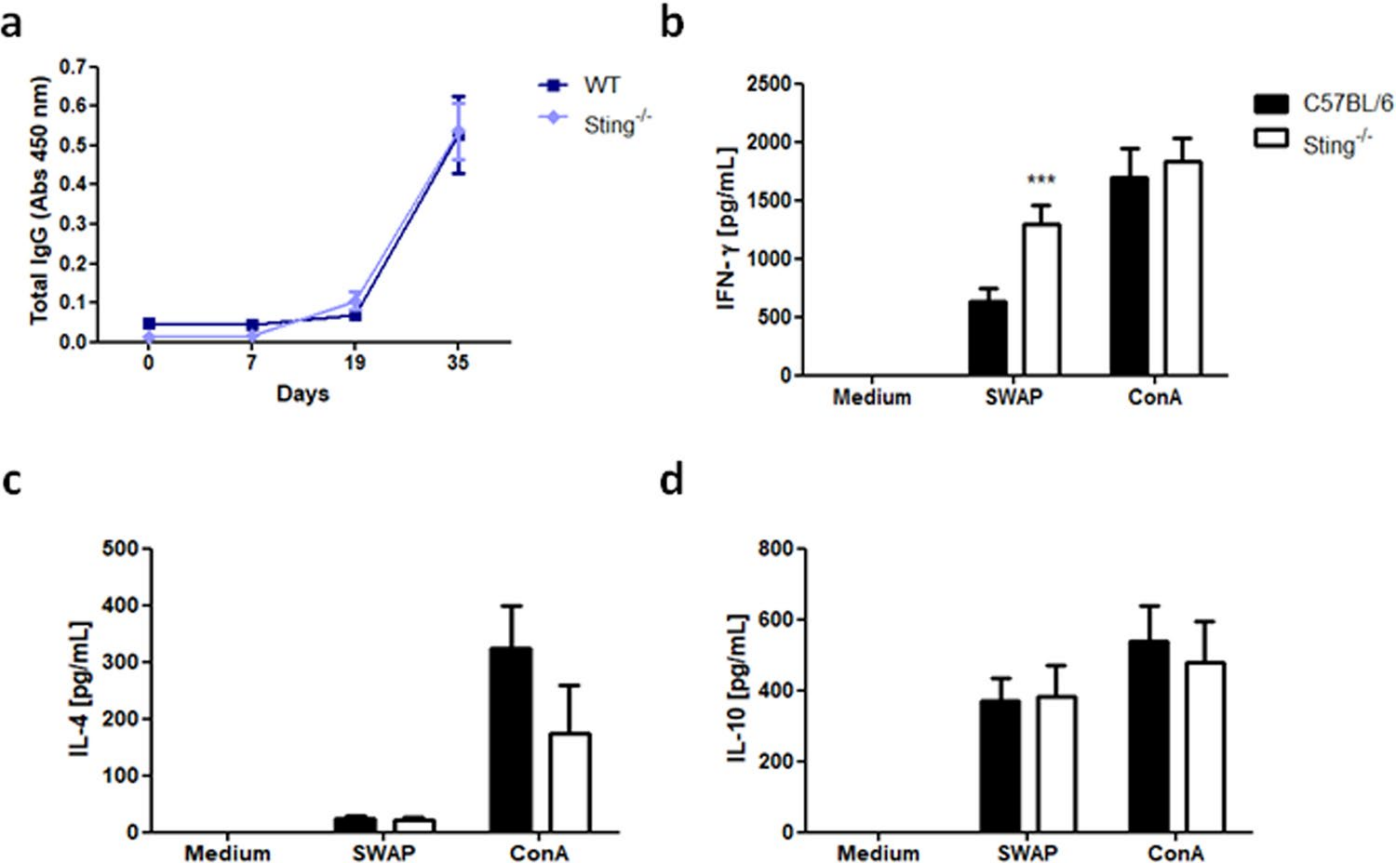
Antibodies and cytokines produced by infected Sting^−/−^ mice. C57BL/6 (WT) and Sting^−/−^ mice were infected with *S. mansoni* cercariae and on days 0, 7, 19, and 35 after infection total IgG anti-SWAP was measured (n = 5) **(a)**. After 40 days of infection, spleens cells were isolated and stimulated with 200 μg/mL of soluble adult worm antigen (SWAP), medium (negative control) or Concanavalin A (ConA) (positive control). Culture supernatants were tested for IFN-γ (n = 5) (**b**), IL-4 (n = 5) (**c**), and IL-10 (n = 5) (**d**) cytokine measurements. (***) is used to demonstrate statistical differences with p < 0.001 compared to the WT mice. Two-Way ANOVA with Bonferroni adjustments were included for multiple comparisons.

Following Sting^−/−^ and WT mice infection with *S. mansoni* cercariae, blood samples were collected to evaluate total IgG anti-SWAP (soluble adult worm antigen) titers in mice sera. As shown in Fig. 3a, there is no significant difference in antibodies produced by Sting^−/−^ and WT mice when evaluated on days 0, 7, 19, and 35 after infection. In order to investigate whether the absence of STING affected cellular immune responses against *S. mansoni*, spleen cells from Sting^−/−^ or WT mice at 40 days of infection were isolated and *in vitro* stimulated with SWAP, medium (negative control) or ConA (positive controls). SWAP-stimulated Sting^−/−^ splenocytes showed significant increase in IFN-γ production compared to WT cells (Fig. 3b). However, no differences were observed in IL-4 and IL-10 production (Fig. 3c,d) in cells from both mouse strains. These findings suggest that *S. mansoni* infected Sting^−/−^ mice presented higher IFN-γ-mediated immune response when compared to WT mice.

### Sting^−/−^ mice present more neutrophils in bronchoalveolar lavage (BAL), lungs, and spleens

**Figure 4:**
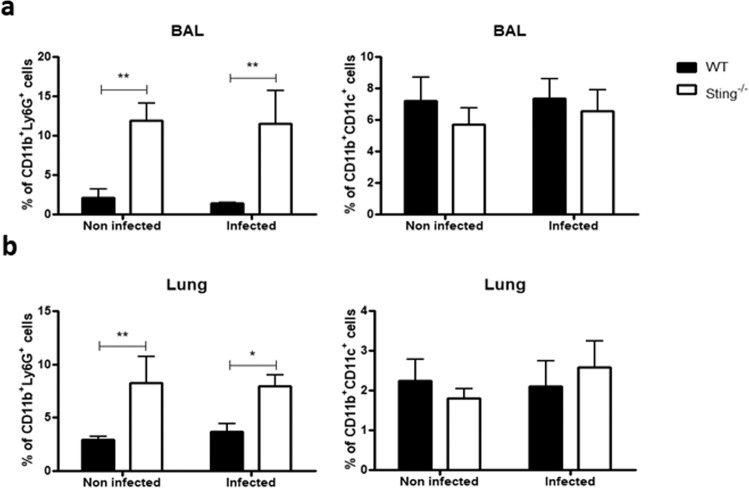
Analysis of percentage of neutrophils and dendritic cells in bronchoalveolar lavage (BAL) and lungs. C57BL/6 (WT) and Sting^−/−^ mice were infected with *S. mansoni* cercariae and after 13 days of infection neutrophils (CD11b+ Ly6G+) and dendritic cells (CD11b+ CD11c+) were quantified in bronchoalveolar lavage (BAL) (n = 5 ) (**a**) and lungs (n = 5) (**b**). (*) or (**) are used to demonstrate statistical differences at p < 0.05 or p < 0.01 compared to the WT mice, respectively. Two-Way ANOVA with Bonferroni adjustments were included for multiple comparisons.

**Figure 5:**
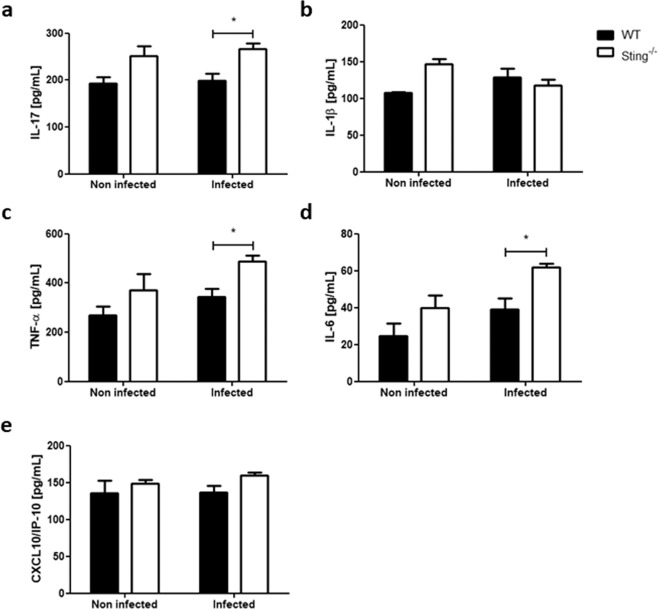
Cytokine profile in BAL of *S. mansoni* infected Sting^−/−^ mice. C57BL/6 (WT) and Sting^−/−^ mice were infected with *S. mansoni* cercariae and after 13 days of infection, the BAL of these animals were collected for IL-17 (n = 5) (**a**), IL-1β (n = 5) (**b**), TNF-α (n = 5) (**c**), IL-6 (n = 5) (**d**), and CXCL10/IP-10 (n = 5) (**e**) cytokine measurements. (*) is used to demonstrate statistical differences with p < 0.05 compared to the WT mice. Two-Way ANOVA with Bonferroni adjustments were included for multiple comparisons.

**Figure 6:**
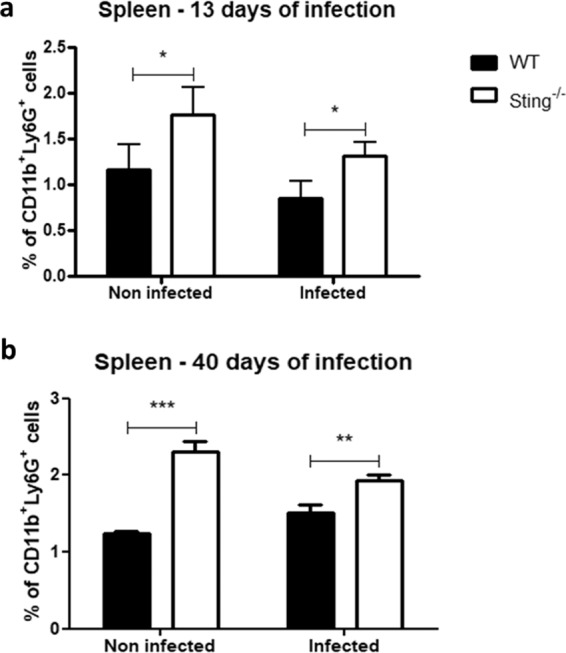
Percentage of neutrophils in spleens of *S. mansoni* infected Sting^−/−^ mice. C57BL/6 (WT) and Sting^−/−^ mice were infected with *S. mansoni* cercariae and the neutrophils (CD11b+ Ly6G+) were quantified in spleen after 13 days (n = 5) (**a**) or 40 days (n = 5) (**b**) of infection. (*) or (**) or (***) are used to demonstrate statistical differences at p < 0.05 or p < 0.01 or p < 0.001 compared to the WT mice, respectively. Two-Way ANOVA with Bonferroni adjustments were included for multiple comparisons.

There is evidence demonstrating that schistosomula become stationary/slow targets for immune attack when they attempt to migrate through the pulmonary capillaries^19^. The cellularity of Sting^−/−^ mice BAL and lungs were evaluated after 13 days of infection in order to determine whether the resistance to infection observed in these mice could also be related to immune attack of the parasite in the lungs. Uninfected Sting^−/−^ mice showed elevated percentage of neutrophils (CD11b+ Ly6G+) in BAL and lungs compared to WT mice. Surprisingly, this increase remains regardless of infection (Fig. 4a). However, no difference was observed in the percentage of dendritic cells (CD11b+ CD11c+) between either infected or uninfected mouse groups (Fig. 4b). WT and Sting^−/−^ mice BAL and lungs were also collected after 13 days of infection for cytokine measurement. No differences were observed in IL-17, IL-1β, TNF-α, IL-6, and CXCL10/IP-10 levels in the BAL between both uninfected mouse groups (Fig. 5). However, after infection Sting^−/−^ mice showed increased levels of the inflammatory cytokines IL-17, TNF-α, and IL-6 (Fig. 5a,c,d) when compared to the WT animals. No difference was observed in cytokine measurements in lungs between infected and uninfected mouse groups (Supplementary Fig. S2). In order to determine whether increased neutrophil population was also observed in another Sting^−/−−/−^ mouse organ, their spleens were also collected after 13 or 40 days of infection for assessment. Uninfected Sting^−/−^ spleen cells showed elevated percentage of neutrophils (CD11b+ Ly6G+) when compared to WT mice, and this increase also remains after infection (Fig. 6a,b). Taken together, these findings suggest that higher frequency of neutrophils in BAL, lungs, and spleens was intrinsic of Sting^−/−^ mice. Besides, BAL of infected Sting^−/−^ mice showed increased inflammatory cytokine profile compared to WT mice.

### Absence of STING results in increased neutrophil survival after IFN-γ stimulation

**Figure 7:**
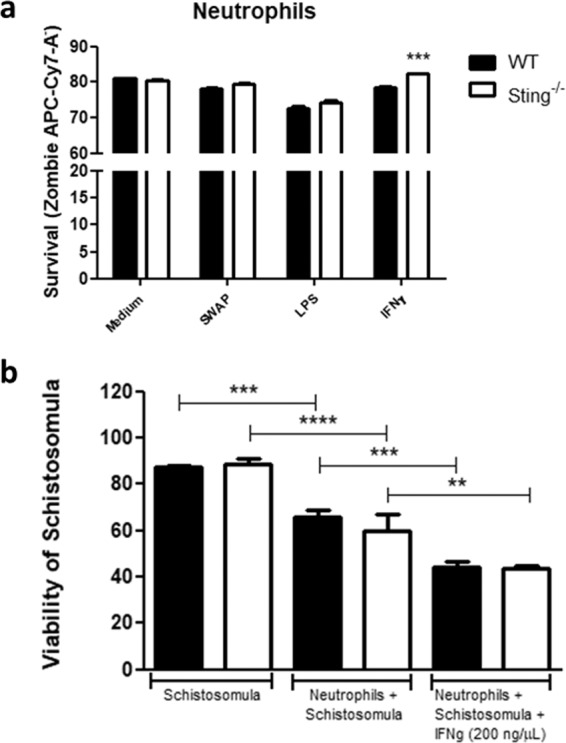
Survival and function analysis of neutrophils after IFN-γ stimulus. Neutrophils from C57BL/6 (WT) and Sting^−/−^ mice were stimulated with 200 ng/μL of IFN-γ for survival evaluation (n = 5) (**a**). Soluble adult worm antigen (SWAP), medium, and lipopolysaccharide (LPS) were used as experimental controls. Additionally, we determine neutrophils ability to kill 100 schistosomula *in vitro* (n = 5) (**b**). (*) or (**) or (***) or (****) are used to demonstrate statistical differences at p < 0.05 or p < 0.01 or p < 0.001 or p < 0.0001, respectively. One-Way (**b**) or Two-Way (**a**) ANOVA with Bonferroni adjustments were included for multiple comparisons.

Neutrophils release reactive oxygen species (ROS), granule lysosomal enzymes and pro-inflammatory cytokines when stimulated with IFN-γ^20^. In order to evaluate IFN-γ effect in the function of these cells against schistosomes, bone marrow neutrophils from WT and Sting^−/−^ mice were cultivated and stimulated *in vitro* with 100 schistosomula. Neutrophils from both mouse groups enhanced similarly their ability to kill the parasites *in vitro* after stimulation with IFN-γ (Fig. 7b). However, neutrophils from WT and Sting^−/−^ mice without exogenous IFN-γ stimulation had reduced ability to kill schistosomula *in vitro* (Fig. 7b). Neutrophils play an important role in the control of schistosomiasis^21^. It was shown that the IFN-γ promotes neutrophils survival by inducing anti-apoptotic mechanisms in these cells^22,23^. In order to evaluate IFN-γ effect in these cells survival, bone marrow neutrophils from WT and Sting^−/−^ mice were stimulated and analyzed by flow cytometry. No differences were observed in neutrophil survival rate of unstimulated and stimulated cells with SWAP or LPS. Surprisingly, Sting^−/−^ mice neutrophils showed increased survival rate when activated with IFN-γ compared to WT mice neutrophils (Fig. 7a).

### Infected Sting^−/−^**mice** displayed inflammatory effects of gut microbiota

**Figure 8:**
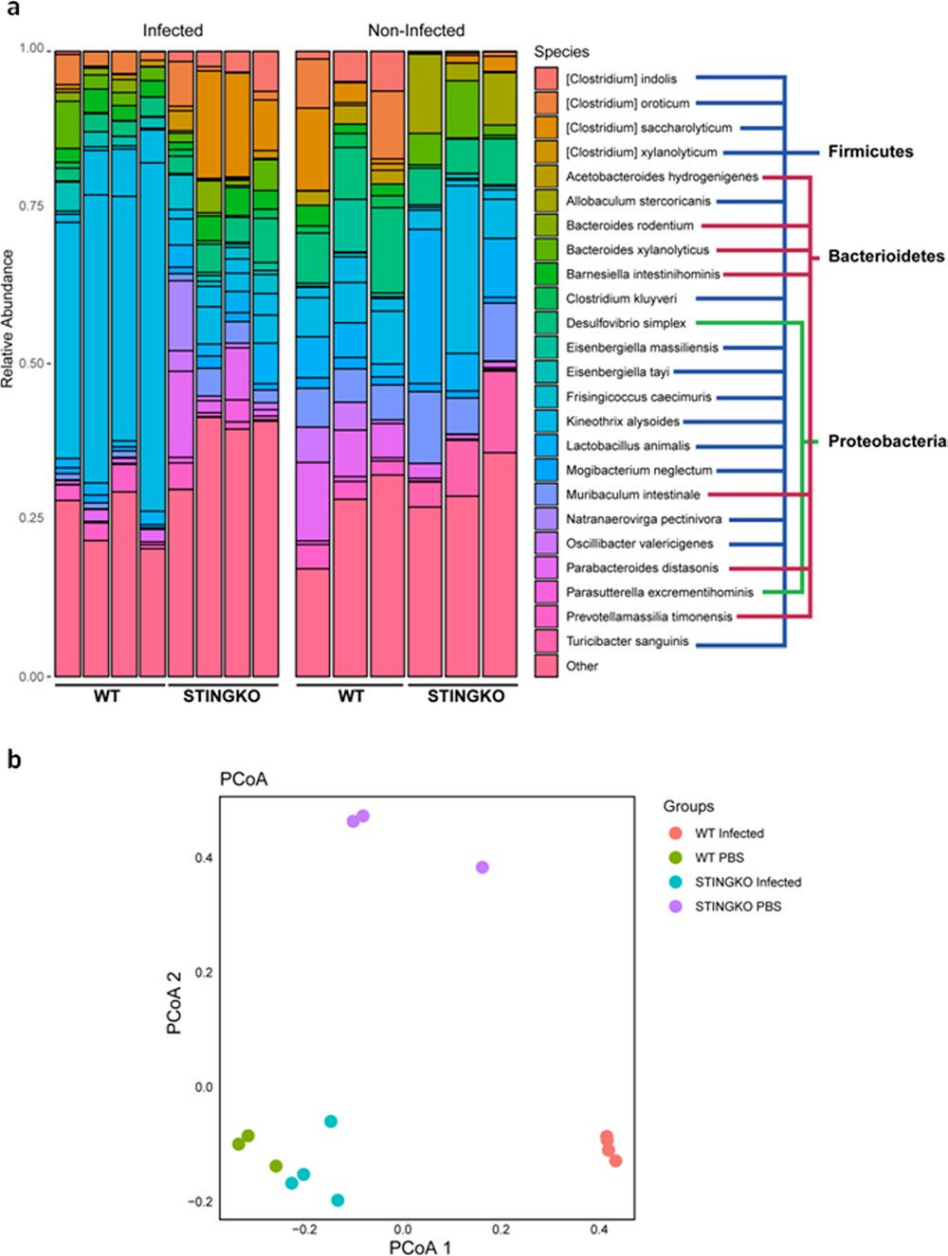
Gut microbiota analysis. C57BL/6 (WT) and Sting^−/−^ mice uninfected (n = 4) or infected with *S. mansoni* cercariae at 40 days (n = 4) were evaluated by the distribution of operational taxonomic units of microorganisms present in feces (**a**), and the structure of microbial communities by unweighted UniFrac Principal coordinate analysis (PCoA) plot for gut bacteria sequenced (**b**). Statistical analysis was performed using the phyloseq^59^. package for R.

Gut microbiota influences many aspects of host physiology, including resistance to infections and immune system development^24^. In order to evaluate whether the absence of STING signaling would interfere in gut microbiota composition; and therefore, in *S. mansoni* infection, feces from all portions of the gut of infected and uninfected WT and Sting^−/−^ mice were collected for 16S RNA sequencing. Beta diversity was assessed by the main coordinate analysis (PCoA) based on unweighted Unifrac distances. Figure 8b shows that uninfected Sting^−/−^ and infected WT mice were clustered separately, indicating significant differences in microbiota composition. Surprisingly, infected Sting^−/−^ and uninfected WT mice presented similarity in gut bacterial population, demonstrated by the proximity of the clusters. Diversity analysis on the microbial communities showed the presence of bacteria belonging to the phyla Firmicutes, Bacteroidetes, and Proteobacteria in the feces (Fig. 8a). However, significant gut microbiota differences between infected and uninfected Sting^−/−^ and WT mice were observed in specific species within these phyla. Infected and uninfected Sting^−/−^ mice presented higher amount of *Desulfovibrio simplex* (Gram-negative) when compared to infected WT mice. Members of the genus *Desulfovibrio* have been related to damage of gut barrier and development of inflammation^25^. Infected Sting^−/−^ mice also presented overgrowth of *Parabacteroides distasonis* (Gram-negative) when compared to the infected WT animals. *P. distasonis* has been associated with gut inflammation for having been found in considerable amount in the Crohn’s disease^26^ and dextran sulfate sodium (DSS)-induced colitis^27^. In contrast, uninfected Sting^−/−^ mice exhibited higher amount of *Lactobacillus animalis and Allobaculum stercoricanis* species that have been previously reported to induce beneficial immunoregulatory effects on gut^28,29^. In summary, analysis of microbiota composition in Sting^−/−^ mice revealed that infection with *S. mansoni* led to an alteration in microbiome composition in these animals towards a more inflammatory profile.

## Discussion

Schistosomiasis is considered the most important human helminth infection in terms of morbidity and mortality worldwide^30^. Due to the great relevance of this disease, a better comprehension of the mechanisms by which the immune system fight against the invading parasite and how it evades host defense responses is required. Recent data have demonstrated that infection by helminths of the *Schistosoma* genus affects the expression of several nucleic acids sensors, such as AIM2, TLR3, and TLR7^5,31,32^. TLR9 signaling pathway was also associated with the regulation of Th2 cytokine–driven granulomatous response^33^. However, the involvement of nucleic acid sensors in schistosomiasis is still poorly understood and needs further investigation.

STING plays an essential role in host defense mechanisms against viral, bacterial and eukaryotic pathogens, but also contributes to the establishment and/or severity of some infections. In a previous study performed with *Plasmodium yoelii*, the mouse model of lethal malaria, the parasite recognition by macrophages was STING-mediated and it induced low levels of type I IFN that primed plasmacytoid dendritic cells (pDCs). This priming, in turn, induced the production of higher amounts of type I IFN, causing a potent inflammatory response and consequent decrease in infected mice survival rate^34^. Supporting these data, another study showed that the activation of cGAS-STING-IRF3-dependent type I IFN signaling leads to a lethal phenotype of *P. yoelii* infection^35^. Additionally, a study performed with *Toxoplasma gondii* demonstrated that the co-expression of cGAS and STING promotes the recognition of parasitic DNA, as well as TBK1 and IRF3 phosphorylation and IFN-stimulated genes (ISGs) induction. The effector ISGs assists in efficient *T. gondii* replication in cell cultures and in mice^36^. In the present study, we demonstrated for the first time that STING can sense *S. mansoni* DNA through cGAS, leading to subsequent increase in *IFN-β* mRNA levels. Surprisingly, STING deficient mice presented higher resistance to infection and significant increase in IFN-γ production by SWAP-stimulated spleen cells when compared to WT mice. High levels of IFN-γ have been associated with the development of acquired resistance against murine schistosomiasis^37^. There is evidence showing a cross-talk between of IFN-αβ and IFN-γ signaling pathways. The IFN-αβ produced in response to an infection down-regulates the IFN-γ receptor (IFNGR) 1 expression on myeloid cells. The decreased expression of this receptor reduces the stimulation of T cells and the subsequent IFN-γ production^34,38^. It has been demonstrated that the optimal protection to *S. mansoni* infection was related to the induction of both B lymphocyte- and IFN-γ-dependent effector immune components^39^. Therefore, it is possible that higher levels of IFN-γ observed in Sting^−/−^ mice may have contributed to the resistance phenotype observed in the present study.

During the progression of schistosomiasis, the schistosomula arrives in the pulmonary vasculature by the venous blood flow between days 2 to 7 after infection. In this phase of the disease, the immunological attack against the parasites become more effective^19^. Among immune cells, neutrophils are the first cell type to arrive at inflammatory sites, being essential in the development of an effective immune response^40^. In helminth infections, neutrophils cause damage to the parasite through the direct contact and/or mediate the recruitment of other effector immune cell populations^41^. In schistosomiasis lung stage, the injury caused on the surface of schistosomula by neutrophils might release tegument particles that can activate surrounding cells^42^. Activated neutrophils release a highly toxic serine protease known as neutrophil elastase (NE). This protease is harmful to *S. mansoni* larvae and adult worms, according to a previous study in which these parasites were sensitive to treatment with NE *in vitro*^21^. Additionally, we have recently demonstrated that *S. mansoni* has developed protective mechanisms to avoid triggering neutrophil activation, such as the expression of a Kunitz-type serine protease inhibitor that acts by blocking NE^40^. In this study, we decided to analyze the neutrophil population in BAL, lungs and spleen of Sting^−/−^ mice. Unexpectedly, we observed that Sting^−/−^ mice present elevated percentage of neutrophils in BAL, lungs and spleen when compared to WT mice. Additionally, enhanced production of IL-17, IL-6, and TNF-α were detected in BAL from Sting-/- mice after infection. Bone marrow neutrophils from WT and Sting^−/−^ mice were also assessed to their ability to kill schistosomula *in vitro* after stimulation with IFN-γ. It was demonstrated that neutrophils from both mice showed the same capacity to kill parasites with or without stimuli. Nevertheless, bone marrow neutrophils from Sting^−/−^ mice showed increased survival rate when stimulated with IFN-γ when compared to neutrophils from WT mice. The association between neutrophils and STING was also reported previously. In the *Staphylococcus aureus* model of cutaneous infection, *Myd88*^−/−^ mice decreased the neutrophil recruitment while Sting^GT/GT^ mice increased neutrophil recruitment and activation leading to restriction of infection^43^. Corroborating with this previous study, the elevated percentage of neutrophils observed in Sting^−/−^ BAL and lungs may correlate with the reduction of *S. mansoni* burden observed in these animals. However, further experiments are required to prove this hypothesis.

Another aspect that may be involved in schistosomiasis infection is the interaction between the immune system and microbiota. It has been described that the balance between inflammatory and anti-inflammatory effects of the gut microbiota has an important role in the establishment/severity of the schistosomiasis in the host^44^. Differences in microbiota composition have also been associated with the granuloma formation and egg migration across the gut wall during *Schistosoma* infection^44,45^. In order to evaluate whether microbiota composition could be interfering in the resistance phenotype of Sting^−/−^ mice to *S. mansoni* infection, the microbiome present in the feces of these mice were identified and analyzed. The clinical relevance of the microbiota composition was demonstrated in a previous study where the shift towards the phylum Proteobacteria was reported in all cases of children with schistosomiasis that presented vomiting, blood in stool and splenomegaly^46^. In this study, the bacteria found in feces of infected and uninfected WT and Sting^−/−^ mice belonged to the Firmicutes, Bacteroidetes and Proteobacteria phyla. Analysis of beta diversity showed that uninfected Sting^−/−^ and infected WT mice presented significant differences in bacterial population composition. Unexpectedly, similarities in infected Sting^−/−^ and uninfected WT mice gut microbiota were demonstrated by the proximity between the clusters. When specific species were evaluated, uninfected Sting^−/−^ mice present lesser amount of *Desulfovibrio simplex* (Proteobacteria phylum - related to the development of gut inflammation) and a higher amount of *Lactobacillus animalis* and *Allobaculum stercoricanis* (both belong to Firmicutes phylum - related to anti-inflammatory effects) compared to uninfected WT. However, after *S. mansoni* cercariae infection, Sting^−/−^ mice presented overgrowth of *Desulfovibrio simplex*, and decrease in *Lactobacillus animalis* and no *Allobaculum stercoricanis* when compared to infected WT. The inflammatory profile of gut microbiota observed in infected Sting^−/−^ mice may have also contributed to the resistance phenotype observed.

In summary, the data presented here demonstrate that the *S. mansoni* DNA is sensed by the cGAS/STING pathway leading to cell activation. Additionally, STING deficient mice infected with *S. mansoni* showed a more pro-inflammatory phenotype featuring increase of IFN-γ production by spleen cells, higher frequency of neutrophils and an inflammatory profile of the gut microbiota that could be some factors related to resistance to infection observed in these animals. This study paves the way for new research on the development of STING inhibitors that could lead to new therapeutic strategies to treat schistosomiasis.

## Methods

### Mice

We used C57BL/6 mice provided from the central animal facility of the Universidade Federal de Minas Gerais (UFMG-Brazil). Mice deficient for STING (Sting^−/−^) and cGAS (cGAS^−/−^) were obtained by Dr. G. Barber (University of Miami-USA)^47,48^. Six-to-twelve weeks of age mice were maintained in isolators at UFMG. All animal experiments were preapproved by the Institutional Animal Care and Use Committee of UFMG (CETEA) under permit #367/2017.

### Parasite (cercariae, Schistosomula, and adult worm)

*Schistosoma mansoni* (LE strain) cercariae were routinely obtained from infected *Biomphalaria glabrata* snails exposed to light, inducing the shedding of parasites at Fundação Oswaldo Cruz - Centro de Pesquisas René Rachou (CPqRR-Brazil). Cercariae numbers and viability were determined using a light microscope, before infection. Schistosomula were obtained after the separation from the tails by centrifugation using a 30% Percoll (Pharmacia, Uppsala, Sweden) solution. Parasites were then cultured for 7 days *in vitro*, as previously described^49^. In order to obtain adult worms, mice were anesthetized with a solution containing 25% Ketamine and 9% Xylazine in 0.9% NaCl and then infected with approximately 100 cercariae through the percutaneous exposure of the abdominal skin for 1 hour. Mice were perfused by the portal hepatic vein after 40 days of infection. Perfusion was performed using phosphate buffer saline (PBS, pH 7.2) containing sodium citrate (15 g/L). Soluble adult worm antigen (SWAP) was obtained by mechanical maceration in PBS. After centrifugation, the supernatant was collected and stored at -80 °C for further assays.

### Worm burden

Protection levels were calculated by comparing the recovered total worm number, total female number and total male number, from each group in relation to the control group. Two independent experiments were performed with n = 10 mice (for each experiment).

### Antibodies

Sera from mice in each experimental group were collected at days 0, 7, 19 and 35 after infection. 96-well microassay plates (Sarstedt, Nümbrecht, Germany) were coated with 20 µg/mL of SWAP in carbonate-bicarbonate buffer (pH 9.6) and then blocked for 2 hours at room temperature with PBS containing 10% heat-inactivated fetal bovine serum (Gibco – Thermo Fisher Scientific, Massachusetts, USA) as previously described^50^. Diluted serum (1:100) was added to each well and plates were then incubated for 1 hour at room temperature. Plate-bound antibodies were detected using peroxidase-conjugated anti-mouse IgG (Sigma-Aldrich, Missouri, USA) diluted 1:2000 in PBS. Color reaction was induced by adding 3,3′,5,5′-Tetramethylbenzidine (TMB) and stopped by adding 5% sulfuric acid to each well. The plates were read at 450 nm in an ELISA reader Multiskan FC (Thermo Fisher Scientific). Two independent experiments were performed with n = 5 mice.

### Liver histopathological analysis

In order to analyze egg numbers, the right lateral lobe of the livers was removed, weighed and dissolved in an aqueous solution of KOH (5%) for 16 hours at 37 °C as previously described^51^. Eggs were then washed in saline and centrifuged twice at 270 x G for 10 minutes and counted under a light microscope. The number of calculated eggs was corrected by considering the mass of the liver, resulting in number of eggs per gram of liver tissue. The reduction levels were calculated by comparing the number of eggs/g of liver of Sting^−/−^ mice in relation to the control group. Liver samples taken from the central part of the left lateral lobe were fixed with 10% PBS buffered formaldehyde. Histological sections of 6 µm were obtained using microtome and stained with haematoxylin-eosin (HE). Granuloma number was counted using a light microscope (10× objective lens) as previously described^50^. Each liver section was scanned to calculate its total area (cm^2^) using ImageJ software (U.S. National Institutes of Health, MD, USA). For the measurement of the granuloma area, images were obtained using a JVC TK-1270/RBG microcamera attached to the microscope (10× objective lens). Twenty granulomas containing a single well-defined egg were randomly selected in each liver section and the granuloma area (mm^2^) was measured using the ImageJ software (http://rsbweb.nih.gov/ij/index.html). Two independent experiments were performed to each liver histopathological analysis with n = 10 mice.

### Spleen cells

Spleen cells were obtained by spleen maceration from individual C57BL/6 and Sting^−/−^ mice after 13 or 40 days of *S. mansoni* infection. Cells were washed with PBS and erythrocytes were lysed with a hemolytic solution (155 mM NH_4_Cl, 10 mM KHCO_3_, pH 7.2). Cells were then adjusted to 1 × 10^6^ in RPMI 1640 medium (Gibco – Thermo Fisher Scientific) supplemented with 10% fetal bovine serum and 1.5% of a solution containing 10000 U penicillin and 10 mg streptomycin/mL per well in a 96-well plate. For cytokine measurement experiments, spleen cells from mice with 40 days of infection were maintained in culture with medium alone (negative control) or stimulated with SWAP (200 μg/mL) or with concanavalin A (ConA) (5 µg/mL). Culture supernatants were collected after 24 hours for IL-4, and after 72 hours for IFN-γ and IL-10 measurements as previously described^50^. Cytokine production was evaluated using the Duoset ELISA kit (R&D Diagnostic, MN, USA) according to the manufacturer’s instructions. Two independent experiments were performed with n = 5 mice. Spleen cells from mice (n = 5) with 13 or 40 days of infection were also analyzed by flow cytometry.

### Bronchoalveolar lavage (BAL)

Tracheas from lethally anesthetized mice were cannulated and the airway lumen washed 3 times (first with 500 μL for evaluation of cytokines and then twice with 500 μL for evaluation of cellularity) with PBS. The first recovered fluids were centrifuged and the supernatants were stored at -80 °C for cytokine analysis. Cytokine production was evaluated using the Duoset ELISA kit (R&D Diagnostic) according to the manufacturer’s instructions. Two independent experiments were performed with n = 5 mice. Cell pellets were resuspended with the recovered fluids for evaluation of cellularity. After a second centrifugation, cell pellets were resuspended in 200 μL of PBS for flow cytometry analysis (n = 5 mice).

### Lung

Fragments of lungs (n = 5 mice) were collected, treated with 20 U/mL of DNAse I (GE Healthcare, Illinois, USA) and 0.13 mg/mL of liberase TL Research Grade (Roche – Sigma-Aldrich) and incubated in a ThermoMixer by 1000 rpm for 45 minutes at 37 °C. Subsequently, the reaction was stopped with RPMI 1640 medium supplemented with 10% fetal bovine serum and 1.5% of a solution containing 10000 U penicillin and 10 mg streptomycin/mL, and filtered through a 70 μm cell strainer. After that, the solution containing the cells were then centrifuged. Cell resuspension was adjusted to 1×10^6^ cells/well for flow cytometry analysis. Fragments of lungs were collected and added 1 mL of cytokines extraction solution [0.4 M NaCl, 0.05% Tween 20, 0.5% bovine serum albumin (BSA), 0.1 mM phenylmethanesulfonyl fluoride (PMSF), 0.1 mM benzethoniumchloride, 10 mM disodium ethylenediaminetetraacetic acid (EDTA) and 20 Kallikrein inhibitor (KI) aprotinin] to each 100 mg of tissue. Then, the Ultra-Turrax homogenizer-dispenser was used to homogenize solutions containing the organs. Subsequently, the samples were centrifuged at 10000 × G for 10 minutes at 4 °C. Cytokine production was evaluated in the supernatant using the Duoset ELISA kit (R&D Diagnostic) according to the manufacturer’s instructions. Two independent experiments were performed with n = 5 mice.

### Flow cytometry analysis

Cells were stained for surface markers. Briefly, the cells were washed and then were incubated for 20 minutes at 4 °C with anti-mouse CD16/32 to block Fc receptors (eBioscience, CA,USA) in FACS buffer (PBS, 0.25% BSA, 1 mM NaN3). After that time, the cells were washed and stained for CD11b, CD11c and Ly6G surface markers for another 20 minutes. Next, the cells were washed and resuspended in PBS as previously described^52^. The events were acquired in Attune Flow Cytometer (Applied Biosystems, CA, USA) and analyzed using FlowJo software (Tree Star, OR, USA). The following reagents were used for staining: FITC-conjugated anti-mouse CD11c (clone HL3, eBioscience), PE-conjugated anti-mouse Ly6G (clone 1A8, eBioscience), APC-Cy7-conjugated anti-mouse CD11b (clone M1/70, BD-Bioscience), and Zombie NIR (BioLegend, CA, USA).

### Confocal microscopy

STING activation was analyzed by immunofluorescence in murine embryonic fibroblasts (MEFs) cells. C57BL/6 MEFs (2.5 × 10^4^) were plated onto 24-well plate containing glass coverslips, and 24 hours later, cells were transfected with *S. mansoni* DNA (3 μg/mL) or dsDNA90 (3 μg/mL) for 6 hours using Fugene Transfection Reagent (Promega, Wisconsin, EUA). Briefly, on the day of transfection, the DNA was diluted in fetal bovine serum-free DMEM medium (Gibco – Thermo Fisher Scientific) containing 0.25% Fugene Transfection Reagent and incubated for 15 minutes. After this time, 100uL of the mix Fugene/DNA was added to each well plate already containing 200uL of DMEM medium with 10% fetal bovine serum. After 6 hours, cells were washed using PBS and fixed in 4% paraformaldehyde for 30 minutes at room temperature. Cells were permeabilized in PBS containing 0.2% Triton X-100 for 10 minutes, blocked for 1 hour with 10% BSA in PBS at room temperature and incubated with a rabbit polyclonal antibody against STING (gifted by Dr Glen Barber, University of Miami, FL) diluted 1:50 in 4% PBS/BSA overnight. Anti-rabbit conjugated with Alexa Fluor 546 (Cell Signaling, MA, USA) diluted 1:500 in 4% PBS/BSA was used for detection of primary antibody. Coverslips were mounted in slides using ProLong Gold with DAPI (4,6-diamidino-2-phenylindole) mounting medium (Invitrogen, CA, USA). All coverslips received the same amount of ProLong Gold with DAPI, in order to cover them completely. Confocal microscopy analysis was performed in a Nikon A1 confocal system. Three coverslips were analyzed per sample and photographs were taken using a 60X objective.

### Quantitative reverse transcriptase–PCR

C57BL/6 (WT) and Sting^−/−^ MEFs non-transfected or transfected with *S. mansoni* DNA or dsDNA90 were homogenized in TRIzol (Invitrogen) and total RNA was isolated in accordance with the manufacturer’s instructions. Reverse transcription of total RNA was performed and Quantitative Real-time RT-PCR was conducted in a final volume of 10 μL containing SYBR Green PCR Master Mix (Applied Biosystems), oligo-dT cDNA (PCR template), and primers. The PCR reaction was performed with QuantStudio3 real-time PCR instrument (Applied Biosystems) as previously described^53,54^. The primers were used to amplify a specific fragment corresponding to specific gene targets as follows:

### β-actin

Forward 5′-GGCTGTATTCCCCTCCATCG-3′ and

Reverse 5′- CCAGTTGGTAACAATGCCATGT-3′;

### IFN-β

Forward 5′-GCCTTTGCCATCCAAGAGATGC-3′ and

Reverse 5′-ACACTGTCTGCTGGTGGAGTTC-3′.

All data are presented as relative expression units after normalization to β-actin.

### Neutrophil isolation

Mice were sacrificed and then the femurs and tibias were removed and cleaned. PBS was forced through the bones and bone marrow cells collected were centrifuged at 270 xG for 10 minutes at 4 °C as previously described^40^. After that time, the pellet was resuspended in RPMI 1640 medium (no phenol red, Gibco – Thermo Fisher Scientific) and filtered through a 70 μm cell strainer. Mononuclear cells were separated from the polymorphonuclear cells and erythrocytes by Ficoll-Paque gradient^55^. Briefly, in a tube containing 3 mL of Ficoll, 2.5 mL of cells were added in medium. After that, the tube was centrifuged at 270 xG for 30 minutes at 4 °C. The fraction containing polymorphonuclear leukocytes and erythrocytes was collected and washed 2 times in RPMI 1640. Erythrocytes were lysed with a hemolytic solution (155 mM NH_4_Cl, 10 mM KHCO_3_, pH 7.2)^56^. Cells were then adjusted to 5 × 10^5^ cells (survival) or 1 × 10^6^ cells (culture with schistosomula) in RPMI 1640 medium supplemented with 10% fetal bovine serum and 1.5% of a solution containing 10000 U penicillin and 10 mg streptomycin/mL per well in a 24 or 96-well plate.

### Neutrophils survival

Neutrophils (5 × 10^5^) in a 96-well plate were maintained in culture with medium alone (negative control) or stimulated with SWAP (200 μg/mL), IFN-γ (200 ng/μL) or lipopolysaccharide (LPS) (1 µg/mL) for 2 hours at 37 °C and 5% CO_2_. After that time, plates were centrifuged and cell pellets were resuspended in PBS for flow cytometry analysis.

### Culture of neutrophils with schistosomula

Neutrophils (1 × 10^6^) in a 24-well plate were initially stimulated with IFN-γ (200 ng/μL) for 2 hours. After that, 7-day freshly prepared schistosomula were washed and resuspended in RPMI 1640 medium supplemented with 10% fetal bovine serum and 1.5% of a solution containing 10000 U penicillin and 10 mg streptomycin/mL. Aliquots containing approximately 100 schistosomula were added in the neutrophils culture for 3 hours at 37 °C and 5% CO_2_. Next, plates were inspected under an inverted microscope (Olympus Co., Hamburg, Germany). In each well, the percentage of dead schistosomula was scored. Dead schistosomula appear as an opaque and granular appearance and lacked flame cell activity as previously described^40^.

### Isolation fecal, DNA sequencing of the gut microbiota and bioinformatics

The feces from all portions of the gut were collected and homogenized in a sterile environment (n = 4 mice for each group). Fecal DNA was extracted from samples using the QIAamp DNA Stool Mini Kit (QIAGEN, Hilden, Germany) and amplified at the V3–V4 hypervariable region of RNA ribosomal 16 S. Subsequently, Illumina-MiSeq platform (Illumina, CA, USA) was used for the sequencing, which provided single-end reads with 300 nt in length. Raw single-end fastq files obtained from sequencing were quality checked using fastqc. All sequences having more than 1 expected error per read were filtered. Operational taxonomic units (OTUs) were generated by clustering sequences with a 99% correspondence and chimera sequences were removed using usearch^57^ (v11). Reads were mapped against the OTU reference to generate a matrix of counts. Subsequently, OTU taxonomy and classification were performed with mothur^58^ (v1.40.5) using the greengenes database.

### Statistical analysis

Statistical analysis were performed using the GraphPad Prism software package (GraphPad, CA, USA).

## Supplementary information

Supplementary information.

## Data Availability

All relevant data are within the manuscript and its Supplementary Material.
