# Supplementary material for: The role of the adaptor molecule STING during Schistosoma mansoni infection
Source: Sci Rep. 2020 May 13;10:7901. doi: 10.1038/s41598-020-64788-6 (PMC7220917; doi:10.1038/s41598-020-64788-6)
Supplement: Supplementary file 1 — Supplementary information. [file 41598_2020_64788_MOESM1_ESM.pdf]

The role of the adaptor molecule STING during *Schistosoma mansoni* infection

Cláudia Souza[1,&], Rodrigo C. O. Sanches[1,&], Natan R. G. Assis[1], Fábio V. Marinho[1], Fábio S. Mambelli[2], Suellen B. Morais[1], Enrico G. T. Gimenez[1], Erika S. Guimarães[1], Tiago B. R. Castro[1] & Sergio C. Oliveira[1,2,3,*]

[1]Departamento de Bioquímica e Imunologia, Instituto de Ciências Biológicas, Universidade Federal de Minas Gerais, Belo Horizonte, MG, Brazil; [2]Departamento de Genética, Ecologia e Evolução, Instituto de Ciências Biológicas, Universidade Federal de Minas Gerais, Belo Horizonte, MG, Brazil; [3]Instituto Nacional de Ciência e Tecnologia em Doenças Tropicais (INCT-DT), Conselho Nacional de Desenvolvimento Científico e Tecnológico, Ministério de Ciência Tecnologia e Inovação Salvador, Bahia, Brazil. [&] These authors equally contributed to this work, *Correspondence and requests for materials should be addressed to S.C.O (e-mail: scozeus1@gmail.com).

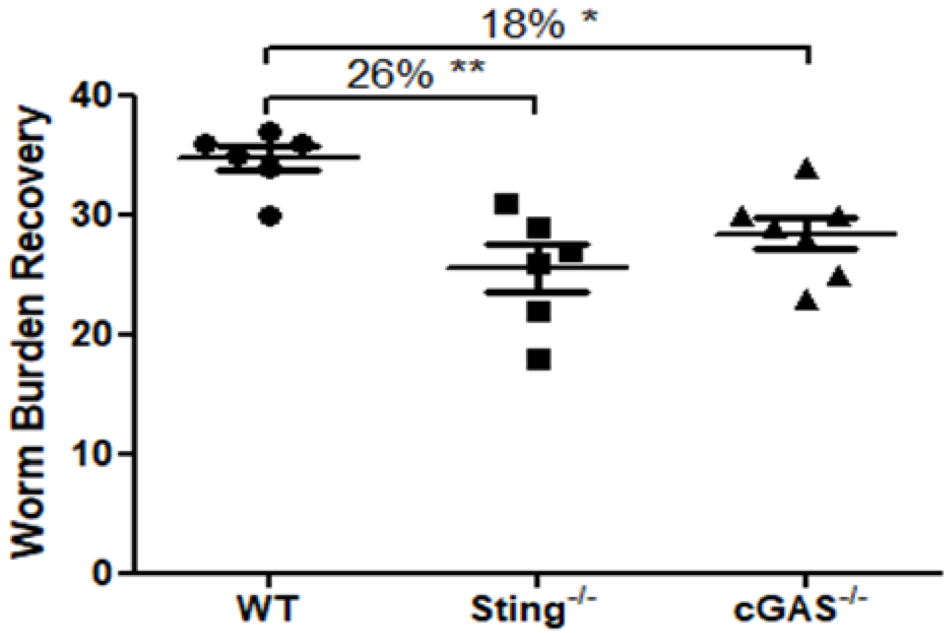

Figure S1. Worm burden recovery in Sting$^{-/-}$ and cGAS$^{-/-}$ mice. C57BL/6 (WT), Sting$^{-/-}$ and cGAS$^{-/-}$ mice (n=10) were infected with 100 *S. mansoni* cercariae and, after 45 days of infection, the total number of worms recovered. (*) and (**) are used to demonstrate statistical differences at $p < 0.05$ or $p < 0.01$ compared to the WT mice, respectively. One-Way ANOVA with Bonferroni adjustments were included for multiple comparisons.

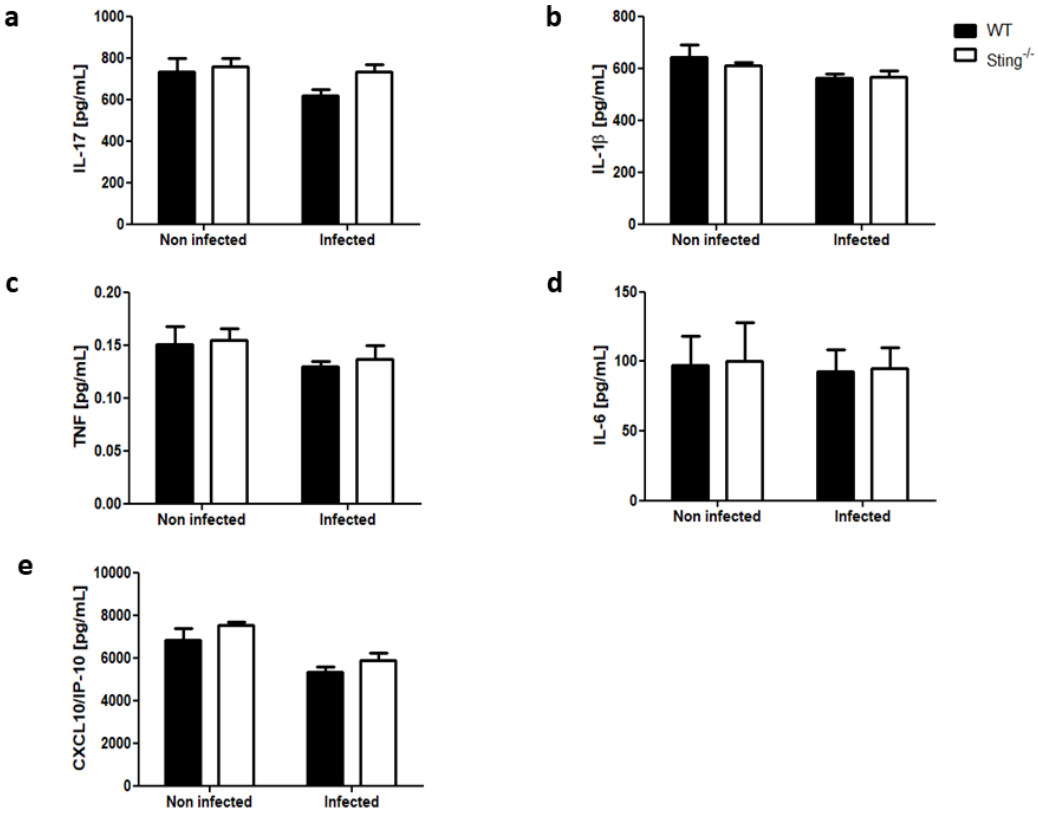

Figure S2. Cytokine profile in the lung. C57BL/6 (WT) and Sting$^{-/-}$ mice were infected with *S. mansoni* cercariae and after 13 days of infection, the lungs were collected, homogenized in cytokine extraction solution and centrifuged. Supernatants were used for IL-17 (n=5) (**A**), IL-1β (n=5) (**B**), TNF-α (n=5) (**C**), IL-6 (n=5) (**D**), and CXCL10/IP-10 (n=5) (**E**) measurements. Two-Way ANOVA with Bonferroni adjustments were included for multiple comparisons.
